# CASC2/miR-24/miR-221 modulates the TRAIL resistance of hepatocellular carcinoma cell through caspase-8/caspase-3

**DOI:** 10.1038/s41419-018-0350-2

**Published:** 2018-02-23

**Authors:** Xiaoxin Jin, Lifeng Cai, Changfa Wang, Xiaofeng Deng, Shengen Yi, Zhao Lei, Qiangsheng Xiao, Hongbo Xu, Hongwu Luo, Jichun Sun

**Affiliations:** 1 Department of General Surgery, The Second Xiangya Hospital, Central South University, Changsha, Hunan 410011 China; 2 Department of General Surgery, The Third Xiangya Hospital, Central South University, Changsha, Hunan 410011 China

## Abstract

Hepatocellular carcinoma is one of the most common solid tumors in the digestive system. The prognosis of patients with hepatocellular carcinoma is still poor due to the acquisition of multi-drug resistance. TNF Related Apoptosis Inducing Ligand (TRAIL), an attractive anticancer agent, exerts its effect of selectively inducing apoptosis in tumor cells through death receptors and the formation of the downstream death-inducing signaling complex, which activates apical caspases 3/8 and leads to apoptosis. However, hepatocellular carcinoma cells are resistant to TRAIL. Non-coding RNAs, including long non-coding RNAs (lncRNAs) and miRNAs have been regarded as major regulators of normal development and diseases, including cancers. Moreover, lncRNAs and miRNAs have been reported to be associated with multi-drug resistance. In the present study, we investigated the mechanism by which TRAIL resistance of hepatocellular carcinoma is affected from the view of non-coding RNA regulation. We selected and validated candidate miRNAs, miR-24 and miR-221, that regulated caspase 3/8 expression through direct targeting, and thereby affecting TRAIL-induced tumor cell apoptosis TRAIL resistance of hepatocellular carcinoma. In addition, we revealed that CASC2, a well-established tumor suppressive long non-coding RNA, could serve as a “Sponge” of miR-24 and miR-221, thus modulating TRAIL-induced tumor cell apoptosis TRAIL resistance of hepatocellular carcinoma. Taken together, we demonstrated a CASC2/miR-24/miR-221 axis, which can affect the TRAIL resistance of hepatocellular carcinoma through regulating caspase 3/8; through acting as a “Sponge” of miR-24 and miR-221, CASC2 may contribute to improving hepatocellular carcinoma TRAIL resistance, and finally promoting the treatment efficiency of TRAIL-based therapies.

## Introduction

Hepatocellular carcinoma, one of the most common solid tumors in digestive system, is a leading cause of cancer-related death worldwide^1^. Despite the achievements in surgery techniques and other therapeutic procedures, the prognosis of patients with hepatocellular carcinoma is still poor due to the acquisition of multi-drug resistance^2,3^. The overall recurrence rate of patients with HCC can reach to over 70%^2,4^; in addition, the 5-year survival rate of patients with stage II HCC is only 50%^5^, indicating that developing novel therapies for HCC has become an urgent need^5^.

TNF related apoptosis inducing ligand (TRAIL), an important ligand of TNF family, can serve as an anti-tumor agent through selectively inducing cancer cell apoptosis but causing no harm to normal cells^6–10^. Several death receptors mediate TRAIL cytotoxicity via the formation of downstream signaling complex which induces cell death, finally activating caspases 3/8 resulting in apoptosis^11–13^. However, the clinic efficiency of TRAIL-based combined therapy is limited due to the acquisition of specific resistance to TRAIL^14–16^. Several cancer cells, such as hepatocellular carcinoma cells, are commonly TRAIL-resistant^17^. Adjuvant agents that can reduce the specific resistance of cancer cells to TRAIL may improve the curative effect of TRAIL-based combined therapy.

In recent years, emerging evidence has regarded non-coding RNAs, such as long non-coding RNAs (lncRNAs) and microRNAs (miRNAs) as major regulators of normal development and diseases, including cancer^18–20^. LncRNAs can serve as “Sponge” of miRNAs to reduce available miRNA activity, thereby preventing miRNAs from binding and negatively regulating their target genes^21^. Under different circumstance, lncRNAs and miRNAs can play a role in tumorigenesis, tumor inhibition or both^22–24^. Moreover, lncRNAs and miRNAs have been reported to be associated with multi-drug resistance^25,26^. Among so far discovered lncRNAs, the tumor suppressive role of CASC2 has been reported in many kinds of cancers^27–29^; in addition, CASC2 is also associated with the chemo-sensitivity of cervical cancer to cisplatin^30^.

In the present study, we monitored the changes in caspase 3/8 in TRAIL-sensitive and TRAIL-resistant hepatocellular carcinoma cells, and searched for candidate miRNAs that might target to regulate caspase 3/8; the expression, role and mechanism of candidate miRNAs in regulating TRAIL resistance of hepatocellular carcinoma cell was then investigated; in addition, we investigated whether CASC2 affected TRAIL resistance of tumor cell through miRNAs. Taken together, we provided a novel experimental theory basis for understanding and treating TRAIL resistance of hepatocellular carcinoma.

## Results

### Screening and identification of candidate miRNAs related to TRAIL resistance of hepatocellular carcinoma

**Fig.1:**
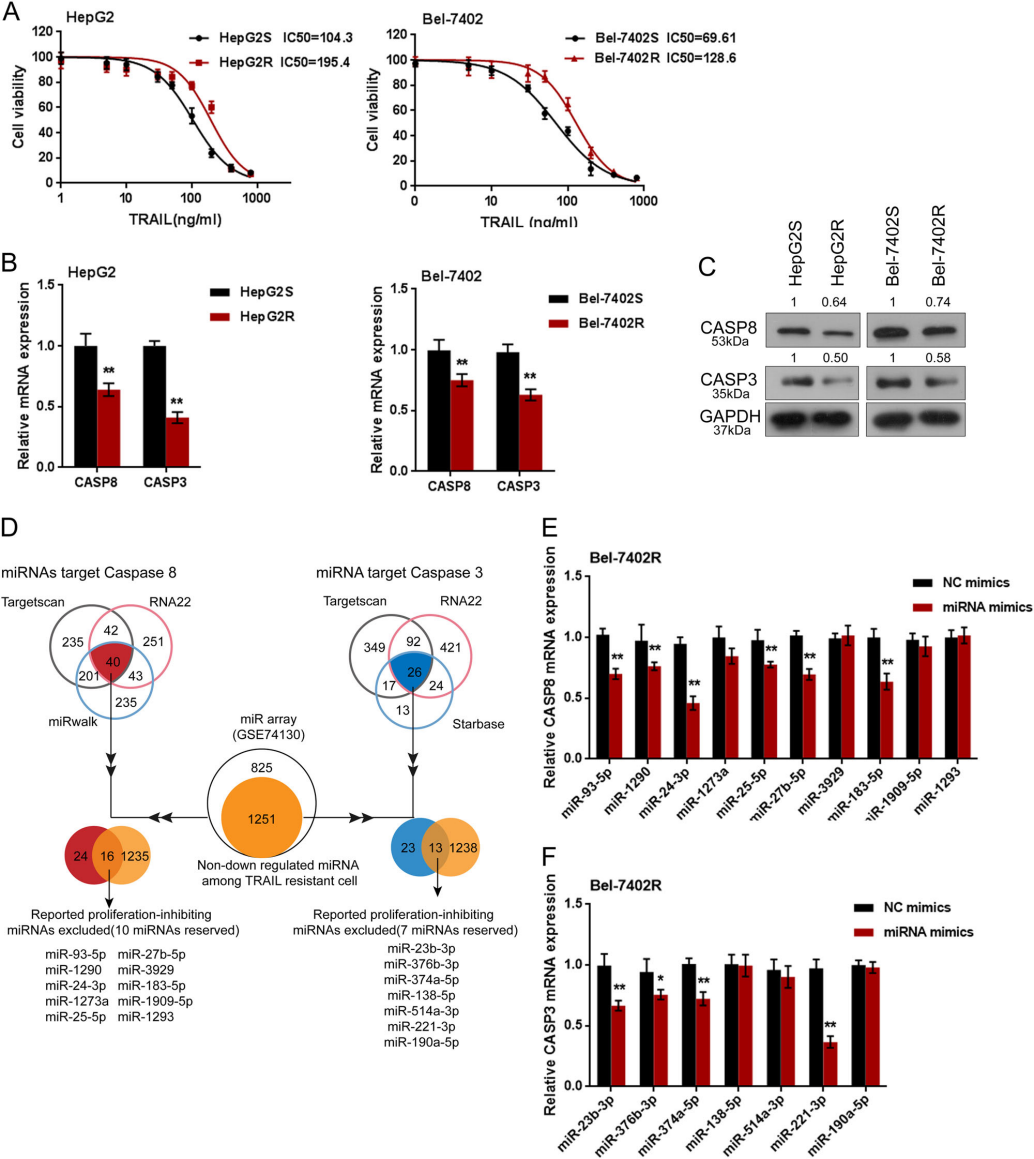
Screening and identification of candidate miRNAs related to TRAIL resistance of hepatocellular carcinoma **a** Regular TRAIL-sensitive HepG2S and Bel-7402S cells (S stands for sensitive) and irregular TRAIL-resistant HepG2R and Bel-7402R cells (R stands for resistant) were treated with a series of doses of TRAIL (1, 10, 100, 1000 ng/ml); the cell viability was determined using MTT assays. Data were shown as a percentage normalized to the viability of cells with no TRAIL treatment. The abscissa was the logarithm of TRAIL concentration (log-conc.). IC50 represented the concentration of TRAIL when cell viability was reduced to 50%. **b** The mRNA expression of caspase 3 and caspase 8 were determined using real-time PCR assays. **c** The protein levels of caspase 3 and caspase 8 were determined using Western blot assays. **d** Online tools (Targetscan, RNA22, miRWalk, and Starbase) were used to search for candidate miRNAs that may regulate caspase 3 or caspase 8, respectively. Non-down-regulated miRNAs in TRAIL-resistant tumor cells were selected in conjunction with the chip data of the GEO database; furthermore, the previously reported miRNAs that inhibited hepatocarcinoma proliferation were excluded according to the literature in NCBI. **e**–**f** Caspase 3 or caspase 8 mRNA expression in response to miRNA overexpression was determined using real-time PCR assays. The data are presented as mean ± SD of three independent experiments. *P < 0.05; **P < 0.01

First, we validated the resistance of hepatocellular carcinoma cell to TRAIL treatment. Regular HepG2S and Bel-7402S cells (S stands for sensitive) and TRAIL-resistant HepG2R and Bel-7402R (R stands for resistant) cells were exposed to a series of doses of TRAIL (1, 10, 100, and 1000 ng/ml); then MTT assays were performed to determine the viability of the cells above. The cell viability of untreated cells was defined as 100%. The results showed that for HepG2S cells, the TRAIL concentration to reduce cell viability to 50% was about 104.3 ng/ml (IC50 = 104.3); for HepG2R cells this value was 195.4 ng/ml (IC50 = 195.4) (Fig.1a). Similar results were observed in Bel-7402S cells, the TRAIL concentration to reduce the cell viability to 50% was about 69.61 ng/ml (IC50 = 69.61), for Bel-7402R cells 128.6 ng/ml (IC50 = 128.6) (Fig.1a).

After death receptor trimerization, TRAIL induces cell apoptosis in a caspase-dependent manner^31^. Herein, we monitored the mRNA and protein expression of caspase 3 and caspase 8 in TRAIL-sensitive and TRAIL-resistant tumor cells using real-time PCR and immunoblotting assays. As shown in Figs.1b, c, the mRNA expression and protein levels of caspase 8 and caspase 3 in TRAIL-resistant HepG2R and Bel-7402R cells were reduced compared to those in the TRAIL-sensitive HepG2S and Bel-7402S cells (Figs.1b, c).

In order to investigate the mechanism by which the TRAIL resistance is affected, we used online tools (Targetscan, RNA22, miRWalk and Starbase) to search for candidate miRNAs that may regulate caspase 3 or caspase 8, respectively. Non-down-regulated miRNAs in TRAIL-resistant tumor cells were selected in conjunction with the chip data of the GEO database; furthermore, the previously reported miRNAs that inhibited hepatocarcinoma proliferation were excluded according to the literature in NCBI (Fig. 1d). MiRNA mimics of 17 selected candidate miRNAs were transfected into Bel-7402R cells to conduct miRNA overexpression, respectively, as confirmed using real-time PCR assays (Fig.S1A-B). Caspase 3 and caspase 8 mRNA expression in response to miRNA overexpression was determined using real-time PCR assays; the results showed that caspase 8 mRNA expression was the most strongly down-regulated by miR-24-3p, while caspase 3 mRNA expression was the most strongly down-regulated by miR-221 (Figs. 1e,f). Thus, miR-24-3p and miR-221 were selected as further research subjects.

### MiR-24/miR-221 promote the resistance of hepatocellular carcinoma cell to TRAIL

**Fig.2:**
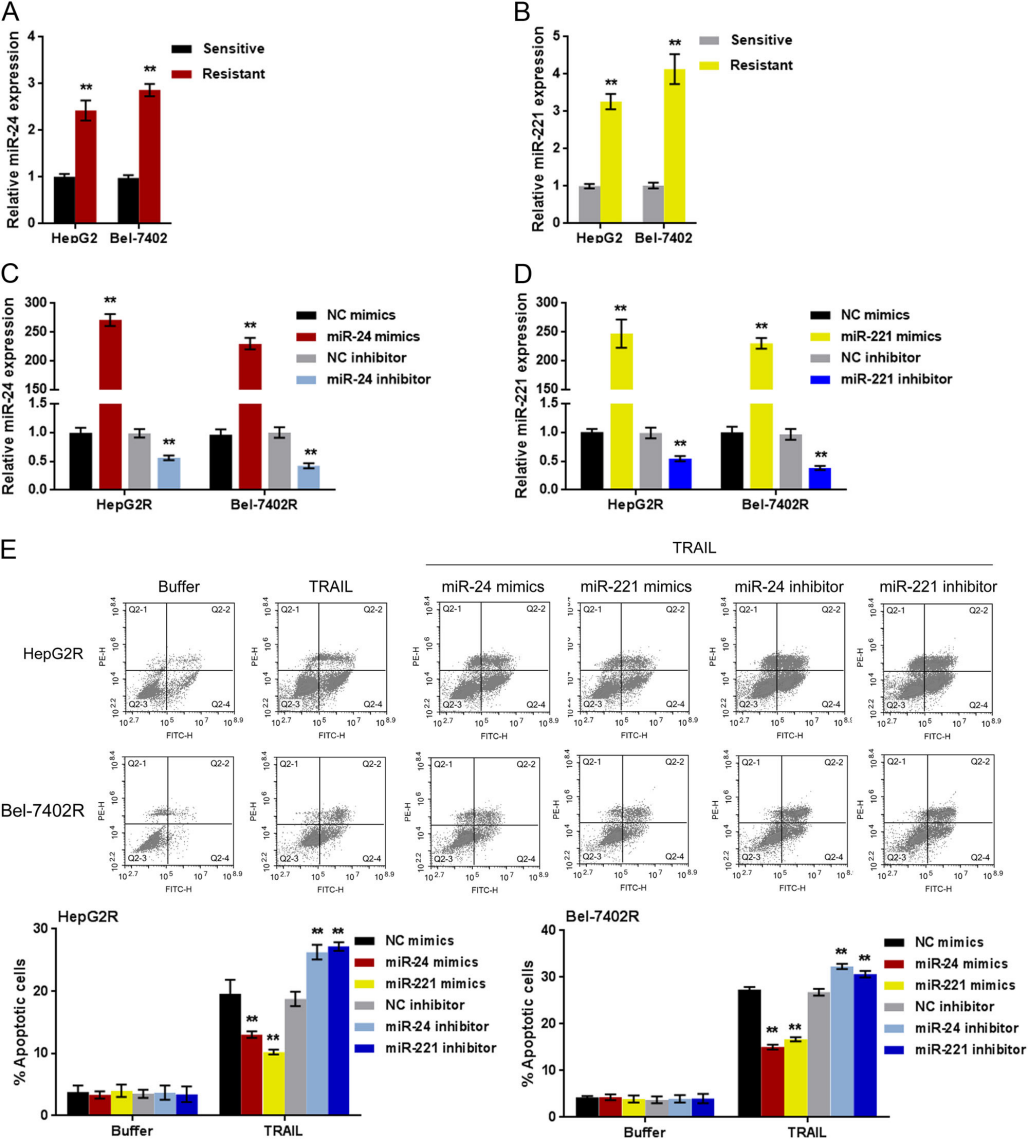
MiR-24/miR-221 promotes the TRAIL resistance of hepatocellular carcinoma cell **a–b** miR-24 and miR-221 expression in TRAIL-sensitive or resistant HepG2 and Bel-7402 cells were determined using real-time PCR assays. **c–d** The mimics or inhibitor of miR-24 and miR-221 was transfected into HepG2R and Bel-7402R cells to achieve miRNA overexpression or miRNA inhibition, as confirmed using real-time PCR assays. **e** The cell apoptosis of the above cells were determined using flow cytometer assays. The data are presented as mean ± SD of three independent experiments. *P < 0.05; **P < 0.01, compared to control group; #*P* < 0.05; ##*P* < 0.01, compared to NC mimics or NC inhibitor group

Next, we investigated the function of miR-24/miR-221 in the resistance of hepatocellular carcinoma cell to TRAIL. In TRAIL-resistant HepG2R and Bel-7402R cells, miR-24 and miR-221 expression was remarkably up-regulated compared to that in HepG2 and Bel-7402 cells (Figs. 2a,b). MiRNA mimics or miRNA inhibitor for miR-24 and miR-221 was transfected into HepG2R and Bel-7402R cells to achieve miRNA overexpression or miRNA inhibition, as confirmed using real-time PCR assays (Figs. 2c,d).

HepG2R and Bel-7402R cells were then transfected with miR-24 mimics/inhibitor or miR-221 mimics/inhibitor in the presence or absence of 100 ng/ml TRAIL treatment; the cell viability and apoptosis were determined using MTT and flow cytometer assays. The results showed that TRAIL treatment significantly suppressed tumor cell viability; the overexpression of miR-24 and miR-221 could promote tumor cell viability, and attenuate the suppressive effect of TRAIL on tumor cell viability; the inhibition of miR-24 and miR-221 could suppress tumor cell viability, and amplify the suppressive effect of TRAIL on tumor cell viability (Fig.S2A-B).

Moreover, TRAIL treatment dramatically increased the apoptosis rate of tumor cell; the overexpression of miR-24 and miR-221 could reduce tumor cell apoptosis rate upon TRAIL treatment, in other words, attenuate the promotive effect of TRAIL on tumor cell apoptosis; on the contrary, the inhibition of miR-24 and miR-221 could increase tumor cell apoptosis rate upon TRAIL treatment, that is, amplify the promotive effect of TRAIL on tumor cell apoptosis (Fig.2e).

### MiR-24/miR-221 negatively regulate caspase 8/3 through direct targeting

**Fig.3:**
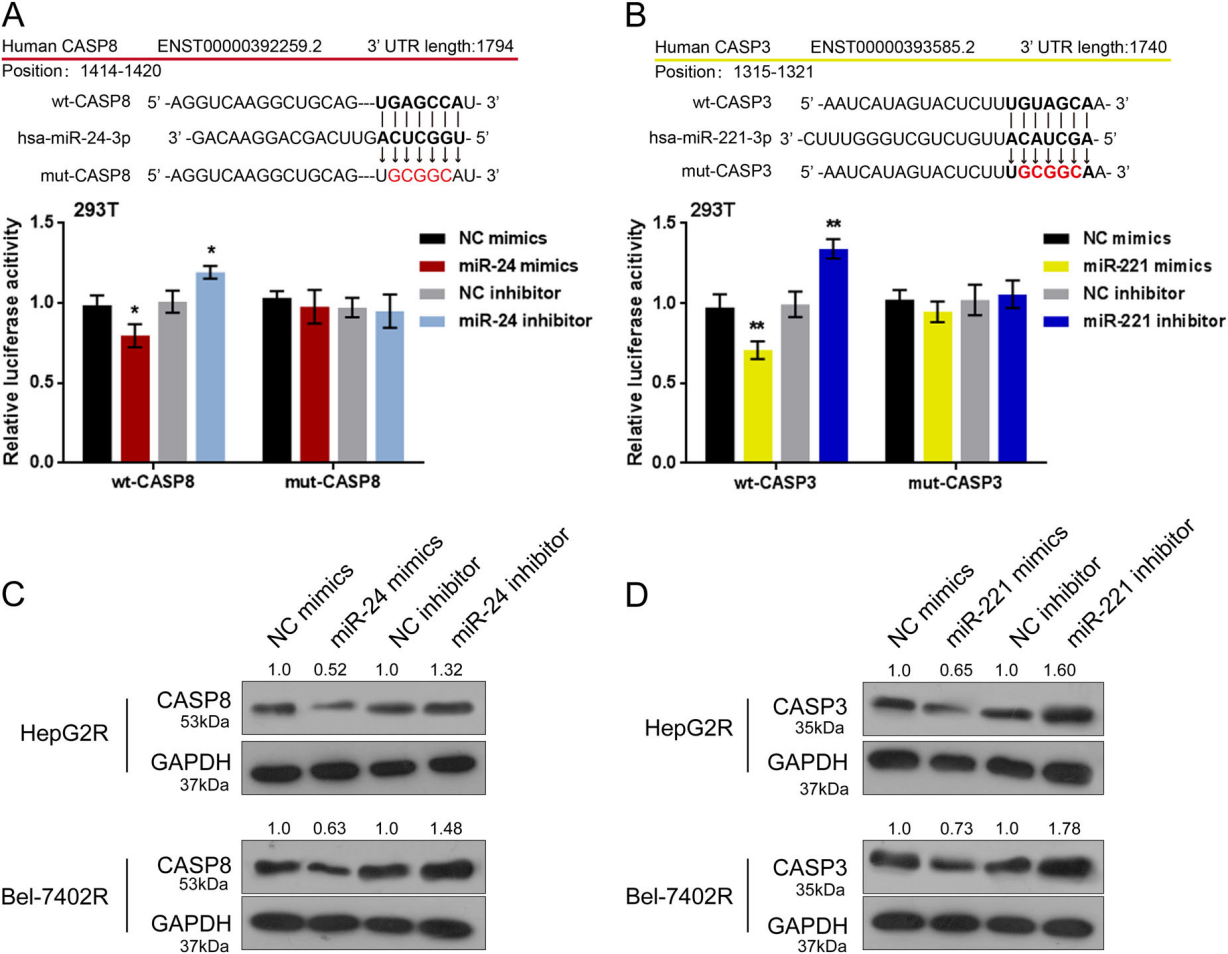
MiR-24/miR-221 negatively regulate Caspase 8/3 through direct targeting **a–b** Wild-type (wt-) and mutated-type (mut-) luciferase reporter gene vectors (wt-caspase 3 named wt-CASP3, wt-caspase 8 named wt-CASP8, mut-caspase 3 named mut-CASP3, mut-caspase 8 named mut-CASP8) were constructed. Mut-CASP3 contains a 5 bp mutation in the predicted miR-221 binding site; mut-CASP8 contains a 5 bp mutation in the predicted miR-24 binding site. The above vectors were co-transfected into HEK293 cells with miR-24/miR-221 mimics or inhibitor; the luciferase activity was determined. **c–d** The mimics or inhibitor of miR-24 and miR-221 was transfected into HepG2R and Bel-7402R cells, respectively; the protein levels of caspase 3 or caspase 8 were determined using Western blot assays. The data are presented as mean ± SD of three independent experiments. *P < 0.05; **P < 0.01

As predicted by online tools, miR-24 may inhibit caspase 8 and miR-221 may inhibit Caspase 3 through targeting. To validate this prediction, luciferase reporter gene assays were performed. Wild-type and mutant-type luciferase reporter gene vectors were constructed, and named wt-CASP8, mut-CASP8, wt-CASP3, and mut-CASP3; mut-CASP8 vector contained a 5 bp mutation at the predicted miR-24 binding site while mut-CASP3 vector contained a 5 bp mutation at the predicted miR-221 binding site (Figs. 3a,b). The above vectors were co-transfected into HEK293T cells with miR-24/miR-221 mimics or inhibitor, respectively. The luciferase activity of wt-vectors was significantly suppressed by miRNA mimics while amplified by miRNA inhibitor; after mutation at the predicted miRNA binding site, the changes of the luciferase activity were abolished (Figs. 3a,b). Next, we validated miRNAs regulation of caspase 3/8 by measurement of caspase 3/8 protein levels. As shown by Western blot assays, Caspase 8 protein was significantly reduced by miR-24 overexpression, while was increased by miR-24 inhibition in HepG2R and Rel-7402R cells (Fig.3c); Caspase 3 protein was also negatively regulated by miR-221 in the above cell lines (Fig.3d). The data confirmed that miR-24/miR-221 negatively regulate caspase 8/3 through direct targeting.

### CASC2 knockdown enhanced TRAIL resistance

**Fig.4:**
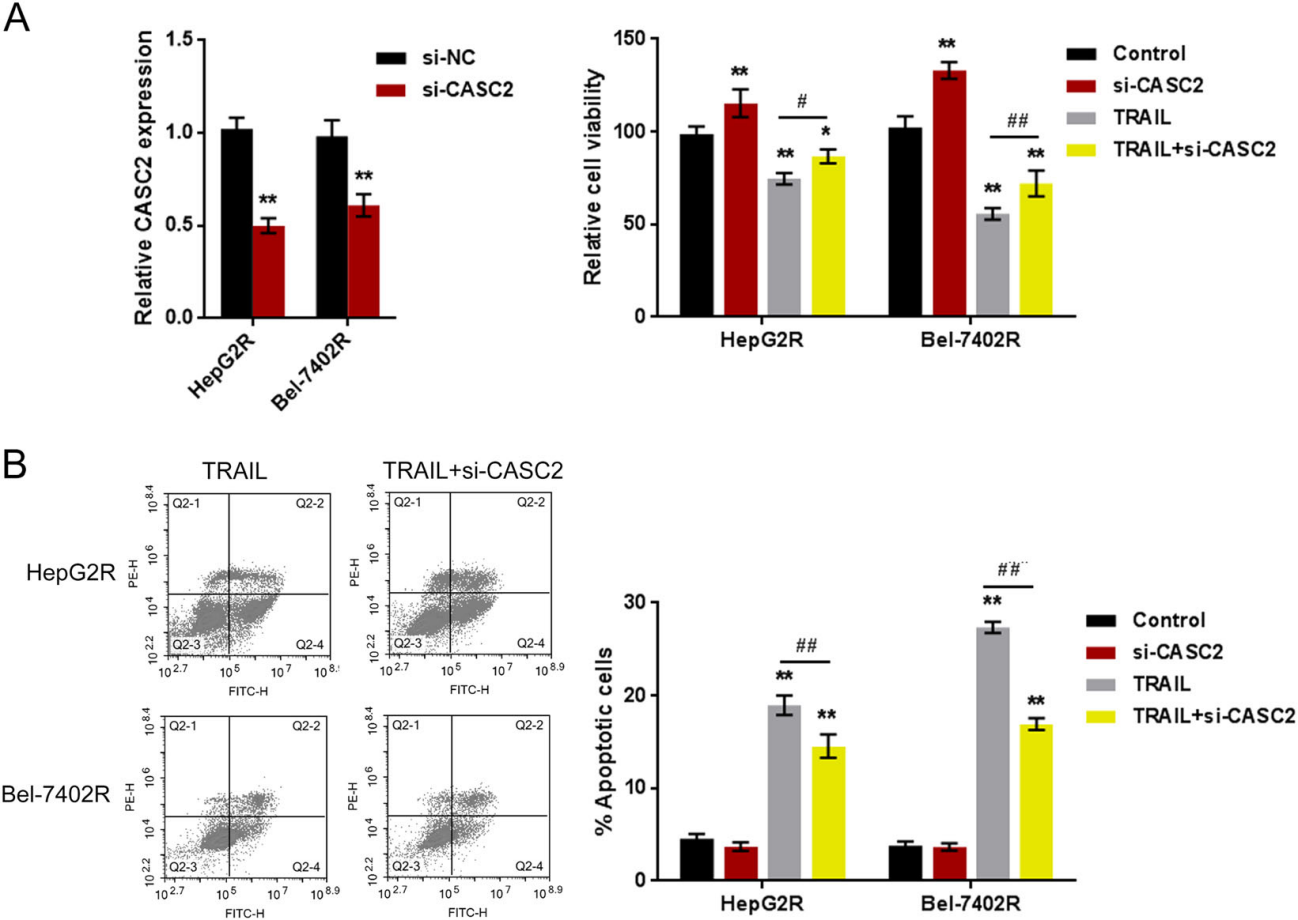
CASC2 overexpression inhibits TRAIL resistance **a** si-CASC2 was transfected into HepG2R and Bel-7402R cells to achieve CASC2 knockdown, as confirmed using real-time PCR assays. Transfected cells were treated with TRAIL; the cell viability was determined using MTT assays. **b** The cell apoptosis was determined using flow cytometer assays (part of the FACS data). The data are presented as mean ± SD of three independent experiments. *P < 0.05; **P < 0.01, compared to control group; ##*P* < 0.01; compared to TRAIL group

CASC2 has been reported to inhibit cancer cell proliferation^32,33^, including hepatocellular carcinoma cell proliferation^34^. Si-CASC2 was transfected into HepG2R and Rel-7402R cells to achieve CASC2 knockdown, as confirmed by real-time PCR assays (Fig.4a). HepG2R and Rel-7402R cells were transfected with si-CASC2, in the presence or absence of TRAIL; the cell viability was determined using MTT assays. The results showed that CASC2 knockdown promoted the cell viability while TRAIL treatment suppressed the cell viability of HepG2R and Rel-7402R cell; the suppressive effect of TRAIL on tumor cell viability could be partially reversed by CASC2 knockdown (Fig.4a). Next, the apoptosis rate of HepG2R and Rel-7402R cell was also monitored. TRAIL treatment promoted cell apoptosis while CASC2 knockdown significantly suppressed the cell apoptosis; the promotive effect of TRAIL on tumor cell apoptosis could be significantly reversed by CASC2 knockdown, indicating the potential role of CASC2 in the TRAIL resistance of HepG2R and Rel-7402R cells.

### CASC2 negatively regulates miR-24 and miR-221 through direct targeting

**Fig.5:**
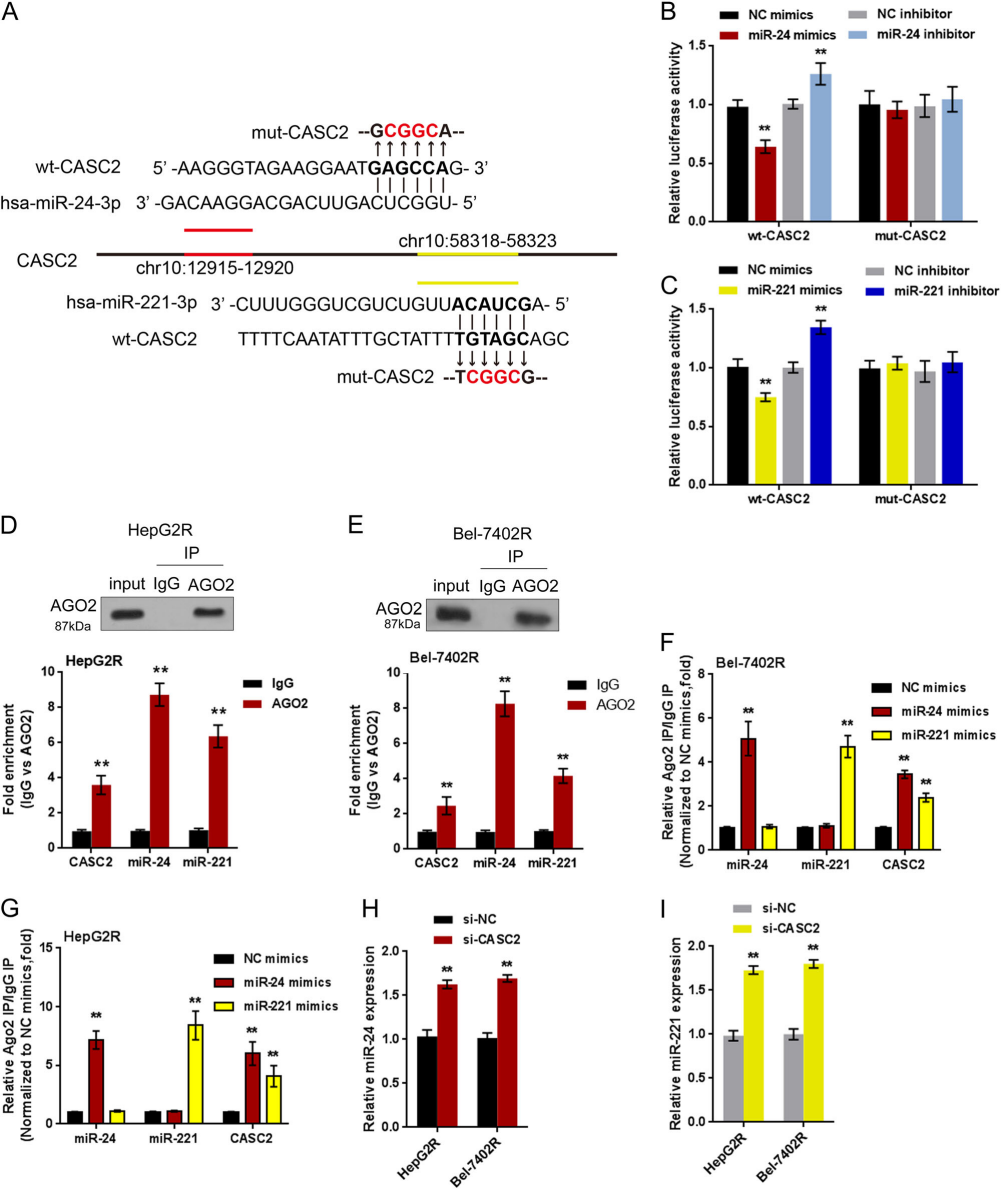
CASC2 negatively regulates miR-24 and miR-221 through direct targeting **a** Wild-type (wt-) and mutant-type (mut-) CASC2 luciferase reporter gene vectors were constructed; the mut-CASC2 vector contained a 4 bp mutation at the predicted miR-24 or miR-221 binding site. **b–c** The indicated vectors were co-transfected into HEK293 cells with miR-24/miR-221 mimics or inhibitor; the luciferase activity was determined. The data are presented as mean ± SD of three independent experiments. **P < 0.01

As predicted by miRCode, CASC2 might bind to miR-24 and miR-221, respectively, to regulate their expression. To validate this prediction and investigate whether CASC2 affects TRAIL resistance through regulating miR-24/miR-221, luciferase reporter gene and RIP assays were employed. Wild-type (wt-) and mutant-type (mut-) CASC2 luciferase reporter gene vectors were constructed; the mut-CASC2 vector contained a 4 bp mutation at the predicted miR-24 or miR-221 binding site (Fig.5a). The vectors were co-transfected into HEK293T cells with miR-24/miR-221 mimics or inhibitor; the luciferase activity was determined. The results showed that the luciferase activity was significantly suppressed by miR-24 mimics or miR-221 mimics, while was amplified by miR-24 inhibitor or miR-221 inhibitor; after mutation at the predicted binding site, the changes of the luciferase activity were abolished (Figs. 5b,c). The data indicate that CASC2 may regulate miR-24/miR-221 expression through direct binding.

To confirm the interaction between CASC2 and miR-24/miR-221, we further performed RIP assay. As shown in Figs. 5d,e, CASC2 and miR-24/miR-221 were associated with the AGO2 in HepG2R and Rel-7402R cells. In RNA extracted from precipitated AGO2 protein, miR-24/miR-221 and CASC2 levels were dramatically higher than IgG. We also performed RIP assay in HepG2R and Bel-7402R cells transfected with control miRNA (miR-NC) or miR-24/miR-221 followed by real-time PCR to detect CASC2 associated with AGO2; the results shown in Figs. 5f,g confirmed the interaction between CASC2 and miR-24, and between CASC2 and miR-221. Moreover, RIP assay of the enrichment of AGO2 on CASC2, caspase 3 and caspase 8 relative to IgG in HepG2R and Bel-7402R cells transfected with CASC2 empty vector (control vector) or CASC2 overexpression vector (CASC2). As shown in Fig.S3A and B, the overexpression of CASC2 leads to the increased enrichment of AGO2 on CASC2, while substantially decreased enrichment on caspase 3 and caspase 8. Combined with the results from luciferase assays, CASC2 competitively binds to miR-24/miR-221 to reduce binding of miR-24/miR-221 to downstream caspase 3/8.

Moreover, CASC2 knockdown significantly promoted miR-24 and miR-221 expression, indicating that CASC2 negatively regulates miR-24/miR-221 expression through direct binding (Figs. 5h,i).

### CASC2 modulates TRAIL resistance through miR-24/miR-221

**Fig.6:**
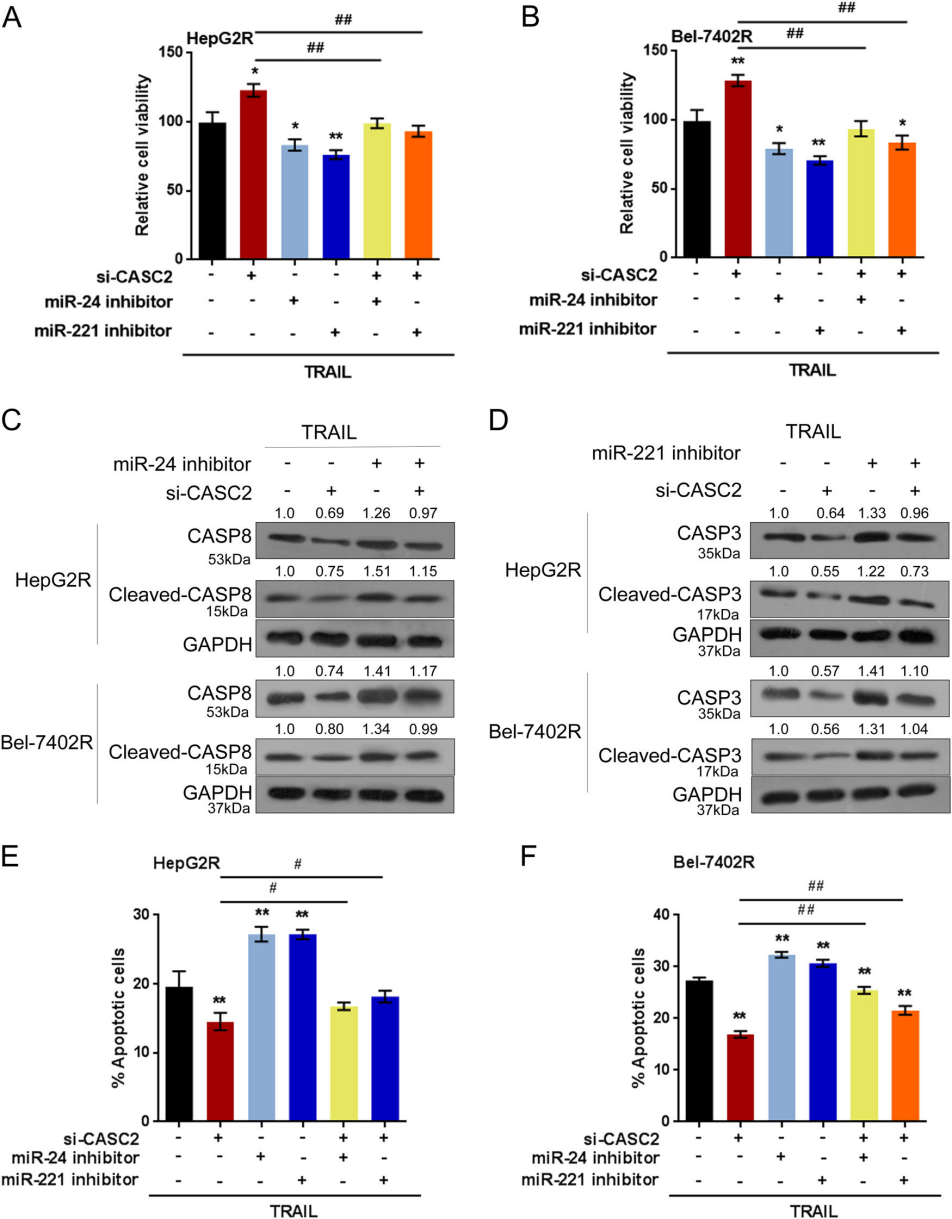
CASC2 modulates TRAIL resistance through miR-24/miR-221 **a-b** HepG2R and Bel-7402R cells were co-transfected with si-CASC2 and miR-24 inhibitor or miR-221 inhibitor upon TRAIL treatment; the cell viability was determined using MTT assays. **c-d** The protein levels of caspase 8 and cleaved-caspase 8 in si-CASC2 and miR-24 inhibitor co-transfected cells, and the protein levels of caspase 3 and cleaved-caspase 3 in si-CASC2 and miR-221 inhibitor co-transfected cells were determined using Western blot assays. **e-f** The cell apoptosis was determined using flow cytometer assays. The data are presented as mean ± SD of three independent experiments. *P < 0.05; **P < 0.01, compared to control group; #*P* < 0.05; ##*P* < 0.01, compared to si-CASC2 group

After confirming that miR-24 directly binds to caspase 8 and miR-221 directly binds to caspase 3, we further validated whether CASC2 modulates TRAIL resistance through miR-24/miR-221. HepG2R and Bel-7402R cells were co-transfected with miR-24 inhibitor/miR-221 inhibitor and si-CASC2 upon TRAIL treatment; the cell viability was then determined using MTT assays. The results showed that si-CASC2 significantly up-regulated tumor cell viability, while miR-24 inhibition and miR-221 inhibition significantly down-regulated tumor cell viability; the promotive effect of si-CASC2 on tumor cell viability could be partially reversed by miR-24 inhibition or miR-221 inhibition (Figs. 6a,b). Next, the protein levels of caspase 8, cleaved-caspase 8, caspase 3 and cleaved-caspase 3 were determined. CASC2 knockdown reduced the protein levels of all the above proteins; miR-24 inhibition promoted caspase 8 and cleaved-caspase 8 proteins and miR-221 inhibition promoted caspase 3 and cleaved-caspase 3 proteins; the inhibitory effect of si-CASC2 on the above proteins could be partially reversed by miR-24 inhibition or miR-221 inhibition (Figs. 6c,d). We also assessed the combined effect of CASC2 knockdown and miR-24/221 inhibition of tumor cell apoptosis upon TRAIL treatment. The results showed that CASC2 knockdown reduced the apoptosis rate of tumor cell; miR-24 or miR-221 inhibition promoted the apoptosis rate; the inhibitory effect of CASC2 knockdown on tumor cell apoptosis could be significantly reversed by miR-24 inhibition or miR-221 inhibition (Figs. 6e,f).

To further confirm the above findings, we searched the Cancer Genome Atlas Liver Hepatocellular Carcinoma (TCGA-LIHC) data collection for the clinical analysis, and revealed that CASC2 expression is down-regulated in the primary tumor, and more down-regulated in advanced stages (Fig.S4A-B and Supplementary Table 1). Higher CASC2 expression causes slight up-regulation in the survival of patients with hepatocellular carcinoma (Fig.S4C); lower miR-24 and miR-221 expression is significantly correlated with longer survival of patients with hepatocellular carcinoma (Fig.S4D-E).

## Discussion

In the present study, we demonstrate that caspase 3/8 protein levels are significantly reduced in TRAIL-resistant hepatocellular carcinoma cells; by using online tools, miR-24 and miR-221 are predicted to target caspase 8 and caspase 3, respectively, to inhibit their expression; in TRAIL-resistant hepatocellular carcinoma cells, miR-24/miR-221 affect TRAIL resistance through direct targeting caspase 8 and caspase 3, respectively; in addition, long non-coding RNA CASC2 affects TRAIL resistance through direct targeting miR-24/miR-221.

TRAIL exerts its apoptosis-inducing effect through four different membrane-bound receptors^35^, among which death receptor 4 (DR4) and death receptor 5 (DR5) are regarded as two apoptosis-inducing receptors^36,37^. Through associating with death-inducing signaling complex, including procaspase-8, DR4 and DR5 are able to deliver apoptosis signal from TRAIL via the functional cytoplasmic death domain^37,38^. In caspase-dependent cell death, procaspase-8 is autocatalytically cleaved or activated, followed by the activation of downstream effector caspases, such as caspase-3 and 7, finally resulting in irreversible cell apoptosis^39^. In the present study, we demonstrated a decrease in caspase 3 and caspase 8 mRNA expression and protein levels in TRAIL-resistant hepatocellular carcinoma cells, suggesting that rescue of caspase 3 and caspase 8 levels may improve TRAIL-resistance in hepatocellular carcinoma.

Up to now, a large amount of miRNAs is reportedly involved in TRAIL-dependent cell apoptosis, representing miRNAs as a promising target to improve the specific resistance to TRAIL-based therapy^40^. So far, studies have identified many miRNAs associating with the specific resistance of hepatocellular carcinoma to TRAIL^41^. Decreased miR-25 expression can enhance the sensitivity of cholangiocarcinoma cell lines to TRAIL-based combined therapy, suggesting the potential role of miR-25 in the specific resistance of cholangiocarcinoma cells to TRAIL^42^. MiR-221 and miR-222 are highly expressed in hepatocellular carcinoma cells compared with that in normal liver cells^43^. Through targeting tumor suppressors phosphatase and tensin homolog (PTEN) and metalloproteinase inhibitor 3 (TIMP3), these two miRNAs are able to induce TRAIL resistance; moreover, MET is also reportedly involved in the activation of miR-221 and miR-222 in non-small cell lung cancer and hepatocellular carcinoma^44^. Based on these previous literatures, we searched several online tools for candidate miRNAs that might target caspase 3 and/or caspase 8 to inhibit their expression in TRAIL-resistant hepatocellular carcinoma cell lines, HepG2R and Bel-7402R. Non-down-regulated miRNAs in TRAIL-resistant tumor cells were selected in conjunction with the chip data of the GEO database; furthermore, the previously reported miRNAs that inhibits hepatocarcinoma cell proliferation were excluded according to the literatures in NCBI. Finally, miR-24 and miR-221 were predicted to target caspase 8 and caspase 3, respectively, to regulate their expression. Subsequently, we conducted a series of functional assays to assess the effect of miR-24/miR-221 on the TRAIL resistance of hepatocellular carcinoma cell. Through direct binding to the 3′UTR of caspase 8, miR-24 overexpression inhibited caspase 8 protein expression, thereby promoting tumor cell viability, suppressing TRAIL-induced cell apoptosis, finally promoting the TRAIL resistance of tumor cell. MiR-221 exerted a similar effect through direct targeting caspase 3. Besides miR-24 and miR-221, several other miRNAs have also been reported to target members of caspase family, thus affecting the cell fate. In cardiomyocytes, caspase 8 has been regarded as a direct target of miR-874 and participates in myocardial necrosis^45^. Bta-miR-29 attenuates the cell apoptosis of Madin–Darby bovine kidney cells through directly targeting caspase 7^46^. The cell apoptosis of bladder cancer cells could be regulated by miR-708 through targeting caspase 2^47^. Herein, we also revealed that miR-24/221 target caspase 3/8 to affect the cancer cell viability and apoptosis, which is consistent with previous studies. Based on this, inhibiting miR-24/miR-221 expression may make a contribution to improving TRAIL resistance.

LncRNAs can serve as “Sponge” of miRNAs to reduce available miRNA activity, thereby preventing miRNAs from binding and negatively regulating their target genes^21^. CASC2, a well-established tumor suppressive lncRNA, is also associated with cancer resistance. CASC2 interacts with miR-181a to modulate the resistance of glioma to TMZ^25^; through regulating miR-21/PTEN axis, CASC2 sensitizes cervical cancer to cisplatin^30^. Herein, we evaluated the role of CASC2 in TRAIL resistance of hepatocellular carcinoma. Consistent with its function in other cancers, CASC2 knockdown significantly promoted tumor cell viability, suppressed tumor cell apoptosis, and increased the resistance of hepatocellular carcinoma cell to TRAIL. As predicted by miRCode, CASC2 could bind to miR-24 and miR-221 to regulate their expression; next we validated whether CASC2 affected tumor cell TRAIL resistance through acting as a “Sponge” of miR-24 and miR-221. As expected, CASC2 negatively regulated miR-24 and miR-221 expression through direct binding. Moreover, the overexpression of CASC2 leads to the increased enrichment of AGO2 on CASC2, while substantially decreased enrichment on caspase 3 and caspase 8. Combined with the results from luciferase assays, CASC2 competitively binds to miR-24/miR-221 to reduce binding of miR-24/miR-221 to downstream caspase 3/8, thereby affecting TRAIL-resistant tumor cell viability, caspase 3/8 protein levels, and finally TRAIL-induced tumor cell apoptosis.

To further confirm the above findings in the present study, we searched TCGA-LIHC data collection for the clinical analysis. CASC2 expression is down-regulated in the primary tumor, and more down-regulated in advanced stages; higher CASC2 expression causes slight up-regulation in the survival of patients with hepatocellular carcinoma, while lower miR-24 and miR-221 expression is correlated with longer survival of patients with hepatocellular carcinoma. These data further indicate that CASC2/miR-24/miR-221 may play a potential role in hepatocellular carcinoma, which helps understand the findings in the present study, and also provides new direction for our future study.

Taken together, we demonstrated a CASC2/miR-24/miR-221 axis, which can affect the TRAIL resistance of hepatocellular carcinoma through regulating Caspase 3/8; through acting as a “Sponge” of miR-24 and miR-221, CASC2 may contribute to improving hepatocellular carcinoma TRAIL resistance, and finally promoting the treatment efficiency of TRAIL-based therapies.

## Materials and methods

### Cell lines and cell transfection

Human hepatocellular carcinoma cell line HepG2 was purchased from the American Type Culture Collection (Manassas, VA, USA); Bel-7402 was purchased from Shanghai Institute of Cell Biology (Shanghai, China). TRAIL-resistant HepG2R and Bel-7402R were purchased from Ori-Bio Biological Technology Co., Ltd (Changsha, Hunan, China). HepG2 was cultured in RPMI-1640 medium (Invitrogen, Carlsbad, CA, USA); Bel-7402R was cultured in HG-Dulbecco’s modified Eagle’s medium (Gibco, Invitrogen). The medium for the culture of cell lines were supplemented with 10% fetal calf serum (PAA, Australia), 100 IU/mL penicillin and 100 IU/mL streptomycin (Amresco, USA). Both cell lines were cultured at 37 °C in a humidified atmosphere consisting of 5% CO_2_ and 95% air.

The overexpression or suppressing expression of miRNA was achieved by transfection of miRNA mimics or miRNA inhibitor (Genepharma, Shanghai, China) using Lipofectamine 2000 (Invitrogen). A small interference RNA of CASC2 (si-CASC2) was used to achieve the knockdown of CASC2 (GeneCopoecia, Guangzhou, China). Cells were plated in 6-well plates or 96-well plates, transfected, incubated for 24 h or 48 h and used for further assays or RNA/protein extraction.

### RNA extraction and SYBR green quantitative PCR analysis

We extracted total RNA from cells using Trizol reagent (Invitrogen, CA, USA) and detected mature miRNA expressions in cells using a Hairpin-it TM miRNAs qPCR kit (Genepharma, Shanghai, China). We used expression of RNU6B as an endogenous control. Caspase-8, 3, and CASC2 expression was measured by the SYBR green qPCR assay (Takara, Dalian, China). Data were processed using 2^-ΔΔCT^ method.

### MTT assay

A modified MTT assay was used to evaluate cell viability. 24 h after seeded into 96-well plates (5000 cells per well), cells were transfected with miRNA mimics or miRNA inhibitor. Medium with or without TRAIL (100 ng/ml) was applied at 24 h post-transfection. 48 h after transfection, 20 μl MTT (at a concentration of 5 mg/ml; Sigma-Aldrich) was added, and the cells were incubated for an additional 4 h in a humidified incubator. 200 μl DMSO was added after the supernatant discarded to dissolve the formazan. OD_490 nm_ value was measured. The viability of the non-treated cells (control) was defined as 100%, and the viability of cells from all other groups was calculated separately from that of the control group.

### Flow cytometer assay

For apoptosis analysis, quantification of apoptotic cells was performed with Annexin V-FITC apoptosis detection kit (Keygen, China). Briefly, the cell samples were harvested with 0.25% trypsin without EDTA after 48 h of infection and then washed twice with ice-cold PBS and re-suspended in 500 μl binding buffer. Then cells were incubated with 5 μl Annexin V-FITC specific antibodies and 5 μl propidium iodide then incubated for 15–20 min in dark and detected by BD Accuri C6 flow cytometer (BD, USA) with the excitation wavelength of Ex = 488 nm and emission wavelength of Em = 530 nm. Each experiment was repeated three times in triplicate.

### Western blot analysis

The protein levels of caspase-3, cleaved-caspase-3, caspase-8, and cleaved-caspase-8 in hepatocellular carcinoma cells were detected by performing immunoblotting. We lysed cultured or transfected cells in RIPA buffer with 1% PMSF, and loaded protein onto an SDS-PAGE minigel and transferred them onto PVDF membrane. The blots were probed with the following antibodies: anti-caspase-3 (ab13585, Abcam, MA, USA), anti-cleaved-caspase-3 (ab2302, Abcam), anti-caspase-8 (C4106, Sigma-Aldrich, St. Louis, MO, USA), anti-cleaved-caspase-8 (C4733, Sigma-Aldrich) at 4 °C overnight, the blots were subsequently incubated with HRP-conjugated secondary antibody (1:5000). ECL Substrates was used to visualize signals (Millipore, MA, USA). GAPDH was used as an endogenous protein for normalization.

### RNA immunoprecipitation

RNA immunoprecipitation assays were performed by using the Imprint RNA Immunoprecipitation Kit (Sigma, St. Louis, USA) along with the AGO2 antibody (Cell signaling, Rockford, USA). The AGO2 antibody was then recovered by protein A/G beads. CASC2, miR-24 and miR-221 levels in the immunoprecipitates were measured by qRT-PCR.

### Luciferase reporter assay

HEK293 cells (ATCC) were seeded into a 24-well plate. Wild-type (wt-) and mutated-type (mut-) luciferase reporter gene vectors (wt-caspase 3 named wt-CASP3, wt-Caspase 8 named wt-CASP8, wt-CASC2, mut-caspase 3 named mut-CASP3, mut-Caspase 8 named mut-CASP8 and mut-CASC2) were constructed. After cultured overnight, cells were co-transfected with the indicated vectors and miRNA mimics or miRNA inhibitor, respectively. Luciferase assays were performed 48 h after transfection using the Dual Luciferase Reporter Assay System (Promega, WI, USA).

### Statistical analysis

Data were exhibited as mean ± SD of three independent experiments and processed using SPSS 17.0 statistical software (SPSS, Chicago, IL, USA). By using the Student’s paired test we compared the differences between two groups. The differences between more than two groups were evaluated using the one-way ANOVA. *P*-values of <0.05 were considered statistically significant.

## Electronic supplementary material

Supplementary Table 1
