# Supplementary material for: CASC2/miR-24/miR-221 modulates the TRAIL resistance of hepatocellular carcinoma cell through caspase-8/caspase-3
Source: Cell Death Dis. 2018 Feb 23;9(3):318. doi: 10.1038/s41419-018-0350-2 (PMC5833678; doi:10.1038/s41419-018-0350-2)
Supplement: Supplementary file 1 — Supplementary Table 1 [file 41419_2018_350_MOESM1_ESM.docx]

**Table-1 Expression of miR-221, miR-24 and CASC2 in normal and liver tumor tissue**

| **Probe set** | **Dataset** | **Tissue** | **Min** | **Q1** | **Median** | **Q3** | **Max** | **P value** | **Sample n** |
| --- | --- | --- | --- | --- | --- | --- | --- | --- | --- |
| hsa-miR-221 | GSE10694 | Normal | 41934 | 80878 | 192484 | 325481 | 1665090 | - | 78 |
| hsa-miR-221 | GSE10694 | Tumor | 41934 | 414652 | 811639 | 1667674 | 5142912 | 0.001 | 78 |
| hsa-miR-221 | GSE6857 | Normal | 244 | 946 | 1449 | 2011 | 8415 | - | 240 |
| hsa-miR-221 | GSE6857 | Tumor | 294 | 1940 | 3235 | 5734 | 47756 | 0.001 | 240 |
| hsa-miR-221 | TCGA | Normal | 23 | 34 | 42 | 60 | 166 | - | 49 |
| hsa-miR-221 | TCGA | Tumor | 23 | 68 | 106 | 140 | 347 | 0.001 | 49 |
| CASC2 | TCGA | Normal | 0.028 | 0.076 | 0.105 | 0.147 | 0.235 | - | 50 |
| CASC2 | TCGA | Tumor | 0 | 0.051 | 0.092 | 0.18 | 0.457 | 3.3586E-07 | 371 |
| hsa-miR-24 | GSE10694 | Normal | 221551 | 1753742 | 2618198 | 3374187 | 5956121 | - | 78 |
| hsa-miR-24 | GSE10694 | Tumor | 41934 | 1974196 | 3024148 | 3959086 | 5956121 | 0.0149 | 78 |
| hsa-miR-24 | TCGA | Normal | 375 | 580 | 679 | 799 | 1240 | - | 49 |
| hsa-miR-24 | TCGA | Tumor | 371 | 550 | 621 | 826 | 1349 | 0.0422 | 49 |
